# A prospective clinical evaluation of fixed dental prostheses made of metal alloys in patients with cerebral palsy

**DOI:** 10.1002/cre2.378

**Published:** 2020-12-13

**Authors:** Nobuaki Magata, Naomi Tanoue, Terumi Ayuse, Takao Ayuse

**Affiliations:** ^1^ Division of Clinical Physiology Nagasaki University Graduate School of Biomedical Sciences Nagasaki Japan; ^2^ Department of Special Care Dentistry Nagasaki University Hospital Nagasaki Japan

**Keywords:** cerebral palsy, dental prosthesis, epilepsy, fixed partial denture, longevity

## Abstract

**Background:**

In general, the prevalence of caries and other oral and dental issues is increased in patients with disabilities, such as those with cerebral palsy. Providing appropriate dental treatment at a primary dental clinic for patients with cerebral palsy and intellectual disability, among other conditions, is challenging.

**Objective:**

The objective of this study was to evaluate the longevity and investigate the related prognostic factors of fixed prostheses in patients with cerebral palsy.

**Methods:**

The records of 36 cerebral palsy patients were used for collecting and analyzing data. A total of 155 prostheses made from metal alloys were finally included in this study. Annual failure rates were calculated; patient‐ and tooth‐related variables associated with prosthesis failure were assessed by a multivariate Cox‐regression analysis and frailty models to introduce random effects.

**Results:**

The 10‐year prosthesis survival rate was 62% and the 20‐year survival rate was 36%. In terms of prosthesis‐related variables, the type of prosthesis had a significant effect, and the hazard ratio of fixed partial dentures was 2.32 times that of single‐unit crowns. In terms of patient‐related variables, the presence of epilepsy had a significant effect on survival, and the hazard ratio for comorbid epilepsy was 3.76 times that for those without comorbid epilepsy.

**Conclusions:**

Our findings suggested that fixed prostheses placed in patients with cerebral palsy might have a particularly low survival rate in cases with comorbid epilepsy. It might also be important to consider the type and/or design of the prosthesis carefully to ensure a better prognosis.

## 1 BACKGROUND

In general, the prevalence of caries in patients with disabilities is high. Patients who have cerebral palsy (CP) are particularly prone to developing oral diseases, such as caries and periodontitis, as they have difficulty in controlling plaque by themselves, due to the peculiar movement disorder caused by inappropriate muscle tone (Vandal et al., 2018). Dos Santos et al. (2002) reported that children with CP had a high decayed, missing, filled surface (DMFS) score, which indicated previous caries activity and a high plaque index. The strong association between a high caries experience and poor oral hygiene in CP patients has been reported previously (Siqueira et al., 2007; Wyne et al., 2017).

Besides caries, patients with CP have other problems related to the oral cavity. For example, significant tooth wear is often observed (DU et al., 2010; Ortega et al., 2014), since muscle tension leads to peculiar jaw movement and occlusion (Mitsea et al., 2001; Winter et al., 2008). Moreover, the rate of trauma, such as tooth fracture and dislocation, is higher in these patients (Bagattoni et al., 2017; Holan et al., 2005; Rodríguez Peinado et al., 2018). Furthermore, these patients often have unexpected chewing and swallowing, lack of control when using a toothbrush and floss, demonstrate excessive gagging, gingivitis due to seizure medications, have food in their mouths for long periods of time, and bite their lips, tongue, and cheek, and so on, which exacerbate oral problems.

Patients with CP strongly require daily dental care and dental restorative and/or prosthetic treatment in order to prevent and limit the severity of the dental diseases, but involuntary movements and posture abnormalities often interfere with rapid dental treatment and hasten the deterioration of the oral environment (Escanilla‐Casal et al., 2014; Kaufman et al., 1991; Mitsea et al., 2001). To reconstruct occlusion in patients with CP, fixed dental prostheses are often placed, since it is difficult for the patients to attach and detach removable prostheses by themselves. The prognosis of fixed prostheses, however, may be worse in these patients than in healthy patients, given the oral conditions of CP patients. An excessive load may be applied to the teeth in CP patients due to bruxism, falls, and so on, which increases the risk of tooth fracture and/or detachment of a prosthesis (Cardoso et al., 2015).

In addition, approximately half of the patients with CP also have an intellectual disability (ID; Chu & Lo, 2010). It is often difficult to treat patients who have both CP and ID at a primary dental clinic. Dental treatments in patients with CP is therefore often carried out in the Department of Special Needs Dentistry. Specialists in special needs dentistry are better able to grasp the pathogenesis of CP and can perform dental treatment in these patients safely. Particularly, in hospital dentistry, where dental treatments under general anesthesia are possible, dentists can treat all patients with CP, even those who are uncooperative.

The purpose of this study was to investigate factors related to the survival of fixed prostheses placed in patients with CP, as this has not been reported to date. This clinical study was conducted on fixed dental prostheses placed in patients with CP who were referred to the Department of Special Needs Dentistry of a university hospital because their dental treatments at a primary dental clinic was impossible.

## 2 MATERIALS AND METHODS

The research protocol was approved by the Research Ethics Committee of Nagasaki University Hospital, Japan (18021902‐2). Informed consent was obtained in the form of opt‐out on the web‐site and bulletin board at the hospital.

This was a retrospective observational study of hospital records of dental patients seen in the Department of Special Care Dentistry, Nagasaki University Hospital, and consisted of the following two experiments: Experiment 1 was a clinical performance evaluation of fixed prostheses placed under general anesthesia in patients with various types of disabilities, as a pilot study, and Experiment 2 was a clinical performance evaluation of all fixed prostheses placed specifically in CP patients regardless of the type of anesthesia adopted. In this study, only prostheses made with metal alloys were targeted to eliminate the influence of materials. The target population of each experiment was as follows: Experiment 1 involved patients whose fixed prostheses had been prepared and placed under general anesthesia from August 1972 through December 2017, and Experiment 2 involved patients with CP whose fixed prostheses were made and placed from August 1984 through December 2017.

Data were digitally extracted and collated in an Excel data file. Researchers accessed the electronic patient files to check data on all prostheses placed during the observation period while respecting privacy regulations related to the patient files. The inclusion criterion for Experiment 1 was the patients with permanent dentition who had fixed prostheses placed under general anesthesia, and that for Experiment 2 was the patients with permanent dentition and with CP, who had fixed prostheses placed during the specific time periods.

### 2.1 Data collection and variables

For Experiment 1, data from 60 patients with 280 fixed prostheses were collected. All patients were uncooperative during prosthetic treatment due to intellectual disability or other neurocognitive disorders. Eight patients who were transferred to other primary or secondary dental institutions after prosthetic treatment and could not be evaluated in our department were thereafter excluded from the study. Additionally, one patient for whom the necessary data was not listed in the hospital record was excluded. Finally, 233 prostheses of 51 patients were selected for this experiment. The data collection was limited to information about the patient's age at the time of prosthesis placement, sex, and medical history (autism spectrum disorder [ASD], CP, ID, and other diseases).

For Experiment 2, 38 patients with 157 fixed prostheses were collected; two patients for whom data were lacking were excluded. Eventually, 155 prostheses in 36 patients were analyzed. The information evaluated included patient data (sex, medical history, including ASD, ID, comorbid epilepsy, and use of a support facility for persons with disabilities) and prosthesis data (age, type of fixed prosthesis [single‐unit crown, connected crown, or fixed partial denture], dental formula, cement used, attending dentist who treated the patients, and anesthesia method). Regarding the variable use of a support facility, both patients in daycare and those who were living in were considered users. For the variable anesthesia method, it was recorded whether general anesthesia or intravenous sedation was used for the prosthodontic treatments. All this information had been collected at the time of prosthesis placement.

All prosthetic treatments were performed indirectly. Abutment preparation was carried out and an impression was made with silicone rubber impression materials. Provisional restorations were made with acrylic materials. The prosthesis was either a full‐coverage crown, facing crown, or fixed partial denture made from metal alloys. The prostheses were luted using glass‐ionomer cement or resin cement, according to the treating dentist.

### 2.2 Outcomes

For each prosthesis, three dates were recorded: the date of placement of the prosthesis, the date of intervention on the prosthesis (if relevant), and the date of the last check‐up, which was considered as the censoring date. As an outcome, when no intervention was required for the prosthesis during the observation period, the prosthesis was considered a success and was censored on the last checkup date. The following three cases were considered as prosthesis failure: (a) extraction of the abutment tooth of the prosthesis; (b) dislodgement of the prosthesis; and (c) removal of the prosthesis for repair, endodontic treatment, replacement with a new prosthesis, and so on.

### 2.3 Evaluation procedures and statistical analysis

Data were checked twice to avoid duplicates and mistakes, and statistical analyses were carried out using JMP 15 software package (SAS Institute Japan, Tokyo, Japan) and R Studio (Foundation for Statistical Computing, Vienna, Austria). Survival time was defined as the period from luting to prosthesis failure, or the most recent follow‐up for surviving prostheses. For both experiments, the same statistical analysis method was adopted. The age variable was converted into three‐category variables after continuity tests. Fisher's exact tests were performed at first to avoid multicollinearity. Survival analysis was performed using the Kaplan–Meier method to obtain the survival curves and the Mantel‐Cox log‐rank test for all prostheses in Experiments 1 and 2. As many patients had more than one prosthesis, there were more prostheses than patients. To address this cluster effect, the factors associated with failure were assessed by a multivariate Cox regression analysis with shared frailty, which considered that observations within the same group (the patient) were correlated and shared the same frailty. The hazard ratios (HRs) and the respective 95% confidence intervals (CIs) were determined. Only those variables presenting *p*‐values <.25 in univariate analyses were selected for the multivariate analysis. A significance level of 5% was considered for all analyses.

## 3 RESULTS

### 3.1 Experiment 1

**FIGURE 1:**
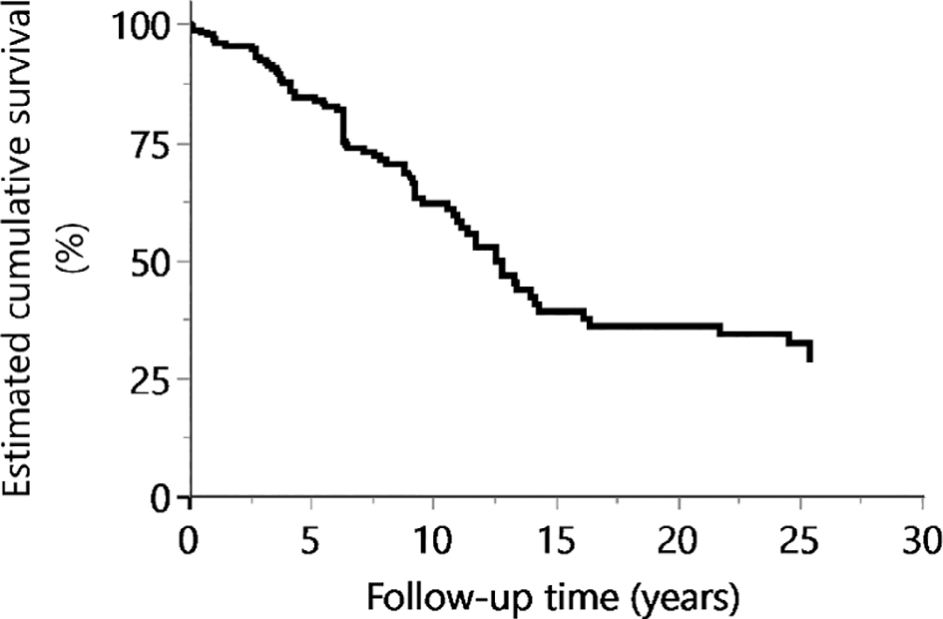
Kaplan–Meier survival curve for all prostheses placed under general anesthesia (Experiment 1)

The longevity of fixed prostheses fabricated and luted under general anesthesia was first investigated, to assess whether the survival of prostheses placed under the same conditions was dependent on the patient's disorder. The distribution of the clinical sample data of Experiment 1 is shown in Table 1. The patients' disability types were ASD (31.4%), CP (15.7%), ID (56.9%), and other diseases (17.6%). Figure 1 shows the Kaplan–Meier survival curve for all prostheses in this experiment. The mean observation time of the prostheses was 7.29 years; the 10‐year survival rate was 62.0% and the 20‐year survival rate was 36.1%. No variable was excluded by Fisher's exact tests, but variable selection using *p*‐values excluded the age and sex variables.

**TABLE 1:** Distribution of fixed prostheses treated under general anesthesia according to tooth‐ and individual‐level variables in Experiment 1 (233 prostheses in 51 patients)

Independent variable	*N*	%	95% CI
Prosthesis‐related variables			
*Age (at time of placement), years*			
Age 1 (≤20)	81	34.8	28.9; 41.1
Age 2 (21–29)	75	32.2	26.5; 38.4
Age 3 (≥30)	77	33.0	27.3; 39.3
Patient‐related variables			
*Sex*			
Male	28	54.9	41.4; 67.7
Female	23	45.1	32.3; 58.6
*ASD*			
Without ASD	35	68.6	55.0; 79.7
ASD	16	31.4	20.3; 45.0
*CP*			
Without CP	43	84.3	72.0; 91.8
CP	8	15.7	8.2; 28.0
*ID*			
Without ID	22	43.1	30.5; 56.7
ID	29	56.9	43.3; 69.5
*Others*			
No	42	82.4	69.7; 90.4
Yes	9	17.6	9.6; 30.3

Abbreviations: ASD, autism spectrum disorder; CI, confidence interval; CP, cerebral palsy; ID, intellectual disability.

The results of the multivariate Cox‐regression analyses with shared frailty, performed after variable selection, are shown in Table 2. In terms of the success of the fixed prostheses, patients with CP showed a higher risk of failure than the other diseases, which was statistically significant, and the presence or absence of ASD, ID, and other diseases did not significantly affect survival. Thus, based on the results of Experiment 1, patients with CP showed a significantly higher HR than those with disabilities such as ASD and ID, for which general anesthesia was also required for prosthesis placement. However, patients with CP consisted of only 15.7% of the subjects of Experiment 1.

**TABLE 2:** Adjusted hazard ratios (HR) for individual‐level variables of Experiment 1 (Cox regression with shared frailty models)

		HR	95% CI	*p*‐Value
ASD (ref = without ASD)				
ASD		2.33	0.95; 5.72	.0650
CP (ref = without CP)				
CP		5.60	1.70; 18.46	.0046
ID (ref = without ID)				
ID		2.17	0.77; 6.09	.1400
Others (ref = without other diseases)				
Other diseases		2.66	0.59; 11.91	.2000
Frailty term				.0410

Abbreviations: ASD, autism spectrum disorder; CI, confidence interval; CP, cerebral palsy; ID, intellectual disability.

### 3.2 Experiment 2

The distribution of the patient‐ and prosthesis‐related variables of Experiment 2 is shown in Table 3. Patients without ID accounted for 63.9%, and this group had a high degree of cooperation in dental treatment. There were few CP patients with ASD (5.6%).

**TABLE 3:** Distribution of fixed prostheses luted in patients with cerebral palsy according to tooth‐ and individual‐level variables of Experiment 2 (155 prostheses of 36 patients)

Independent variable	*N*	%	95% CI
Prosthesis‐related variables			
*Age (at time of placement), years*			
Age 1 (13–28)	55	35.5	28.4; 43.3
Age 2 (29–41)	49	31.6	24.8; 39.3
Age 3 (42–67)	51	32.9	26.0; 40.6
*Categories of anesthesia*			
Awake	72	46.5	38.8; 54.3
Anesthesia	83	53.5	45.7; 61.2
*Cement*			
Resin cement	46	29.7	23.0; 37.3
Other cements	109	70.3	62.7; 77.0
*Dental formula*			
Maxilla	74	47.7	40.0; 55.6
Mandible	81	52.3	44.4; 60.0
Anterior	45	29.0	22.5; 36.6
Posterior	110	71.0	63.4; 77.5
*Operator*			
Main doctor	49	31.6	24.8; 39.3
Other doctors	106	68.4	60.7; 75.2
*Type of prosthesis*			
Single‐unit crown	89	57.4	49.5; 64.9
Fixed partial denture	60	38.7	31.4; 46.6
Connected crown	6	3.9	1.8; 8.2
Patient‐related variables			
*Sex*			
Male	19	52.8	37.0; 68.0
Female	17	47.2	32.0; 63.0
*ASD*			
Without ASD	34	94.4	81.9; 98.5
ASD	2	5.6	1.5; 18.1
*Comorbid epilepsy*			
Without epilepsy	27	75.0	58.9; 86.2
Epilepsy	9	25.0	13.8; 41.1
*ID*			
Without ID	23	63.9	47.6; 77.5
ID	13	36.1	22.5; 52.4
*Use of support facility*			
No	22	61.1	44.9; 75.2
Yes	14	38.9	24.8; 55.1

Abbreviations: ASD, autism spectrum disorder; CI, confidence interval; ID, intellectual disability.

**FIGURE 2:**
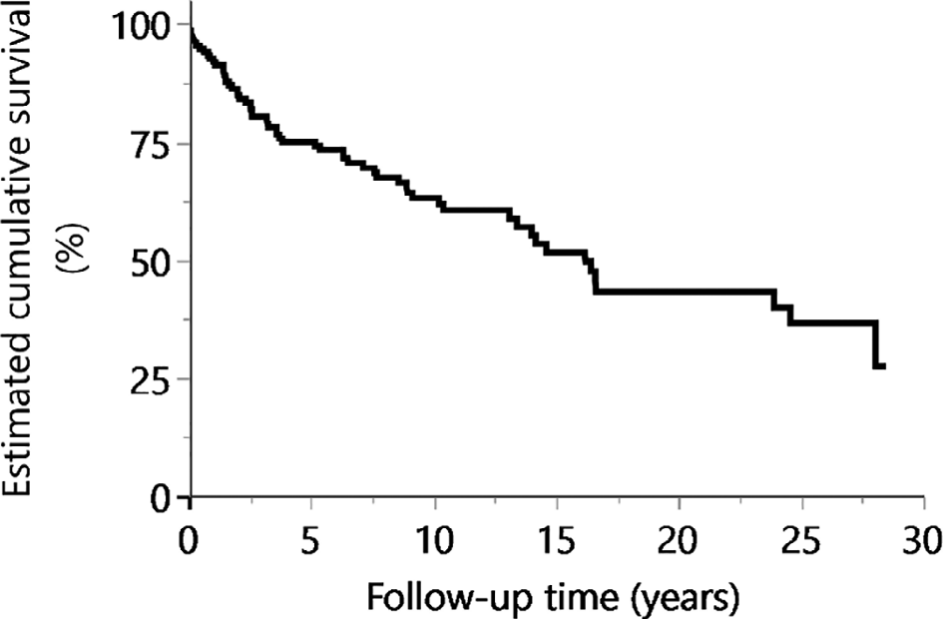
Kaplan–Meier survival curve for prostheses placed in patients with cerebral palsy (CP; Experiment 2)

Figure 2 shows the Kaplan–Meier survival curve for all prostheses set in patients with CP in this experiment. The mean observation time of the prosthesis was 8.28 years; the 10‐year survival rate was 63.2% and the 20‐year survival rate was 43.3%, both of which were higher than the rates observed in Experiment 1. The cement‐type variable was excluded by Fisher's exact tests, and variable selection by p‐values also excluded sex, dental formula, cement, treating dentist, use of a support facility, and anesthesia method from inclusion as variables in multivariate analysis. The multivariate Cox‐regression analyses results performed on the remaining variables are shown in Table 4. Among the remaining variables, the type of prosthesis and comorbid epilepsy variables were significant factors. The HR of fixed partial dentures was 2.32 times that of a single‐unit crown. As a patient‐related variable, the HR for comorbid epilepsy was 3.76 times that for absence of comorbid epilepsy.

**TABLE 4:** Adjusted hazard ratios (HR) for both tooth‐ and individual‐level variables of Experiment 2 (Cox regression with shared frailty models)

	HR	95% CI	*p*‐Value
Age (ref = age 2)			
Age 1	0.83	0.34; 2.04	.6800
Age 3	1.60	0.74; 3.45	.2300
ASD (ref = without ASD)			
ASD	2.38	0.47; 12.06	.2900
Comorbid epilepsy (ref = without epilepsy)			
Epilepsy	3.76	1.17; 12.10	.0260
ID (ref = without ID)			
ID	0.39	0.11; 1.41	.1500
Type of prosthesis (ref = single‐unit crown)			
Connected crown	2.42	0.27; 21.84	.4300
Fixed partial denture	2.32	1.30; 4.15	.0044
Frailty term	.0180		

Abbreviations: ASD, autism spectrum disorder; CI, confidence interval; ID, intellectual disability.

The prosthesis survival rates in Experiment 2 (Figure 2) were higher than those in Experiment 1 (Figure 1), but the log‐rank test result revealed no significant difference between survival curves for the two experiments (*p* = .882).

## 4 DISCUSSION

In Experiment 1, among the patients who required prosthodontic treatments under general anesthesia, only those with CP showed a difference in the survival rate of fixed prostheses depending on the presence or absence of the condition. Consequently, it was considered that the presence of CP could be a factor that affects the survival rate of the prosthesis. Therefore, in Experiment 2, factors affecting the survival rate of the prosthesis were assessed for all patients with CP, regardless of the anesthesia method used.

The overall survival rate in Experiment 2 was higher than that in Experiment 1. This might be because the percentage of patients without ID in Experiment 2 (63.9%, Table 3) was higher than that in Experiment 1 (43.1%, Table 1). Patients without ID might have more accurately understood the importance of daily dental care. Nevertheless, the 10‐year prosthesis survival rate in CP patients based on Experiment 2 (63.2%) was lower than that previously reported for prostheses placed in healthy patients (Behr et al., 2014; Ferrari et al., 2017; Kassardjian et al., 2016; Pjetursson et al., 2015; Reitemeier et al., 2013; Sailer et al., 2018; Walton, 2015). Behr et al. (2014) reported a 10‐year survival rate for single‐unit porcelain‐fused‐to‐metal crowns of 92.3–95.9% in a cohort of healthy participants, and Sailer et al. (2018) reported that the 10‐year survival estimate of fixed prostheses was 100% in a healthy cohort. These high survival rates were probably due to well‐managed daily dental care (Behr et al., 2014).

If the factors that influence the low prosthesis survival rate of CP patients are elucidated, we may be able to bring the survival rates of prostheses in CP patients closer to that of healthy individuals by reducing negative influencers accordingly. Consequently, in our study, the type of fixed prosthesis and the presence of epilepsy were statistically significant predictors of prosthesis failure.

Regarding the type of fixed prosthesis, the HR of fixed partial dentures was higher than that of single‐unit crowns. Generally, it is said that there is no significant difference in the survival rates of a single‐unit crown and fixed partial denture among healthy patients. Concerning the loss of a fixed metal‐ceramic prosthesis or tooth, Reitemeier et al. (2013) reported that the survival rate was 94.3% at 8.0 years for a single‐unit crown and 94.4% at 11.0 years for a fixed partial denture; in addition, the difference between survival functions was not significant. Our finding that the prosthesis structure was related to its longevity might be a particular characteristic of patients with CP.

For a thorough assessment of the influence of the type of prosthesis on survival rate, further statistic sub‐analysis was performed using a logistic regression mixture model (Table 5). The objective variable was the type of prosthesis (single‐unit crown or fixed partial denture), and the explanatory variable was the reason for the failure (extraction, dislodgement, and removal). The results revealed that dislodgement was the most common cause of failure and was unlikely to lead to tooth extraction.

**TABLE 5:** Logistic regression mixture model results between fixed partial dentures by prosthesis type and reason of failure

	*β* (SE)	Wald	OR	95% CI	*p*‐Value
Reason of failure (ref = extraction)					
Dislodgement	1.96 (0.96)	2.05	7.12	1.09; 46.29	.040
Removal	0.52 (0.96)	0.54	1.68	0.25; 11.10	.590

Abbreviations: CI, confidence interval; OR, odds ratio; SE, standard error.

Fixed partial dentures have the disadvantage that abutment teeth tend to become overburdened by the deformation process inside the intermediate section of the construction.(Reitemeier et al., 2019) The mobility of the teeth is generally limited, and although the rigidity of the prosthesis could resist external force, its limited mobility might lead to detachment of the prostheses.(Styranivska et al., 2017) Poor oral hygiene in CP patients might also have had detrimental effects, particularly in terms of the tissue around the pontics. However, the effect of oral hygiene could not be proven since it was not assessed.

As a patient‐related variable, the presence of epilepsy had a significant effect on survival: the HR for CP cases with comorbid epilepsy was 3.76 times that of cases without epilepsy. To assess the influence of epilepsy, further statistics sub‐analysis using a logistic regression mixture model (Table 6) as well as an analysis of the effects of the type of prosthesis were performed. The objective variable was set to with or without epilepsy and the explanatory variable was the reason for the failure. Consequently, there was no association between the presence or absence of epilepsy and the cause of failure, and the results indicated that failure can occur if epilepsy is merged. In this study, it was not possible to elucidate what caused the prosthesis to fail due to the merger of epilepsy. Generally, the occurrence of oral trauma and bruxism may be a representative factor related to the failure of the prosthesis (Reitemeier et al., 2013). Previous studies showed that patients with epilepsy had an increased risk of losing teeth (Djemal et al., 1999) and fracturing crowns (Karolyhazy et al., 2005). Drug‐induced gingival overgrowth is a side‐effect associated with anticonvulsants (e.g., phenytoin); it is quite likely that the condition of periodontal tissue might have affected the oral environment (Gerreth & Gerreth, 2014). Nevertheless, what must be highlighted in this study's analysis is that only the fixed prostheses in patients with epilepsy were at high risk of failure. Further investigations assessing periodontal tissue and stress analyses are warranted to address the issue.

**TABLE 6:** Logistic regression mixture model results between the merger of epilepsy and the reason for failure

	*β* (SE)	Wald	OR	95% CI	*p*‐Value
Reason of failure (ref = extraction)					
Dislodgement	0.22 (5.01)	0.04	1.25	0.00; 2.31e+04	.965
Removal	−0.38 (6.70)	−0.06	0.68	0.00; 3.47e+05	.954

Abbreviations: CI, confidence interval; OR, odds ratio; SE, standard error.

### 4.1 Study limitations

This study had some limitations. There might have been other variables peculiar to patients with CP that were not assessed in this study. The general condition, living environment, and oral environment, including oral hygiene and occlusion may vary greatly in patients with disabilities. As seen in the frailty terms of Tables 2 and 4, significant differences were observed in the variance of random effects, due to variations across patients in the models of both Experiments 1 and 2. This indicates that frailty exists on an individual level.

### 4.2 Clinical implications

Patients with CP must be treated prosthodontically after careful determination of their personality and cooperation. In a previous study, there was no statistically significant difference in tooth morbidity between a group of healthy children and a group of children with CP (Schöpper et al., 2016), but there was a significant association between the frequency of daily care and the subject's level of oral hygiene (Grzić et al., 2011). Starting at an early age, better organization of the preventive dental care are needed to increase the level of dental health of CP patients. In particular, dentists need to pay attention to the design of prostheses and the presence or absence of epilepsy in the patients, which is an important factor that affects the prosthesis prognosis.

## 5 CONCLUSION

The estimated survival rate of prostheses in patients with CP was found to be related to the presence or absence of epilepsy and the type of fixed prosthesis (single‐unit crown or fixed partial denture) used. Prosthetic design should be carefully carried out, particularly in those with comorbid epilepsy.

## CONFLICT OF INTEREST

The authors declare there is no conflict of interest.

## Data Availability

The data that support the findings of this study are available on request from the corresponding author. The data are not publicly available due to privacy or ethical restrictions.
